# Is the perceived public stigma of smokers associated with value opposites? An exploratory cross-sectional analysis of Norwegian data 2011–2013

**DOI:** 10.3389/fsoc.2023.1051189

**Published:** 2024-01-11

**Authors:** Gunnar Sæbø, Marianne Lund

**Affiliations:** Norwegian Institute of Public Health, Oslo, Norway

**Keywords:** public stigma, smoking, tobacco denormalisation, public health ethics, value opposites

## Abstract

Smoker stigma is a likely unintended consequence of tobacco polices aiming to denormalise smoking. Little is known about the dissemination of stigmatising attitudes toward smokers at the population level, including their associations with personal values. Applying a theoretical approach that conceptualises stigma as a cultural (moral and intersubjective) issue, we analyse the spread of perceived public stigma of smokers in Norway and factors predicting agreement with such a perception. Using merged data from the biennial national survey *Norwegian Monitor* 2011 and 2013 (*N* = 7,792), we tested whether the tendency to agree with a perceived public stigma of smokers differs by four indexes of value opposites (‘puritanism/emancipation,’ ‘conformity/individuality,’ ‘tolerance/intolerance,’ ‘status/anti-status’), controlling for smoking status, SES, and demographics. Descriptive statistics and block-wise logistic regression models were applied. In the total sample, 59.1% agree with the statement that ‘most people think less of a person who smokes.’ Two of the four indexes of value opposites tested were associated with tendencies to agree with the perceived public stigma of smokers (‘puritanism/emancipation’ and ‘status/anti-status’). Smokers with current plans to quit expressed the highest perceived public stigma, while ex-smokers expressed a higher perceived public stigma than never-smokers. Women, young people and respondents with high SES agree with a public stigma of smokers more than men, older people and respondents with low SES do. The perceived public stigma of smokers is high in Norway and varies to some extent with personal values, but also with socio-demographics and especially smoking status.

## Introduction

### Tobacco denormalisation and the stigmatisation of smokers

The term tobacco denormalisation designates all policies and interventions that aim to enforce and reinforce the idea that ‘tobacco use is not a mainstream or normal activity in society’ (Lavack, 1999, p. 82). Introduced in tobacco control in the late 1990s to counter the continuous publicity and advertising of the tobacco industry, the idea has not only been to denormalise the act of smoking, but also to delegitimise all tobacco industry activities and tactics (Sæbø and Scheffels, 2017). Examples of denormalisation measures are indoor smoking bans in all public premises, bans on smoking in private cars when children are present, and prohibition of all forms of direct and indirect advertising (including display bans in shops). No other legal consumer product is regulated as strongly as the cigarette to prevent purchase and use, not even alcoholic beverages.

From a public health point of view, tobacco denormalisation has been a successful population-level approach to reducing the prevalence of smoking (Chapman and Freeman, 2008; Malone et al., 2012). However, increasing anecdotal evidence and findings from several, primarily qualitative, studies suggest that tobacco denormalisation has also had the unintended additional consequence of contributing to stigmatise remaining smokers in the population (Evans-Polce et al., 2015; Machado et al., 2018), including unsuccessful quitters (Sæbø and Lund, 2020) and people already stigmatised due to other conditions (Hefler and Carter, 2019; Lipperman-Kreda et al., 2019). Even if there is no manifest intention in tobacco denormalisation strategies to discriminate against or stigmatise people who smoke, tobacco policy measures that implies restrictions on behaviour or environmental restructuring, such as smoke-free air laws, has contributed to the experience of a ‘pillory-like’ situation among smokers outside public premises (Bell et al., 2010; Evans and Furst, 2016). Many smokers are addicted to nicotine and tend to communicate a poor self-image and to perceive themselves in a patronising manner, even when they are not smoking (Ritchie et al., 2010). Smokers are also discredited by non-smokers via derogatory stereotypes (Gibson, 1998; Gilbert et al., 1998). The overtly irrational aspect of smoking is provocative to many non-smokers nowadays and may contribute to a view among the latter group that current smokers, having voluntarily decided to smoke and not listen to information and warnings, should bear the full responsibility for this unhealthy behaviour (McCool et al., 2013). Kim and Shanahan (2003) have launched the concept of ‘unfavourable smoking climate’ to designate the anti-tobacco culture that has developed in the wake of tobacco denormalisation policies. Most likely, this ‘unfavourable smoking climate’ is a result of smoking being a unique type of social action, in that it is still legal, but simultaneously regulated so strongly that it is considered as illegitimate by the social and political majority.

Even if the perceived and felt stigma among different smaller groups of smokers is relatively well-documented in the literature, few studies have addressed the formation and dissemination of stigmatising attitudes toward smokers among never-smokers, and the scope and role of perceived stigmatisation among current smokers and ex-smokers, at the *national* level. Previous studies show a stronger tendency to stigmatise smokers among people who have never smoked than among ex-smokers (Peretti-Watel et al., 2014; Brown-Johnson and Popova, 2016). Such findings corroborate the social ‘us and them’ distance between non-smokers and smokers described above, but also suggest that direct experience with smoking and having (had) a certain social identity as a smoker may have a modifying effect on holding stigmatising perceptions of smokers. This effect may extend to people living with smokers in their household, as they too are less likely to stigmatise smokers (Peretti-Watel et al., 2014).

Apart from the obvious significance of smoking status, however, not much is known about what may predict variations in such attitudes, even if media representations (Bain et al., 2017), age and socio-economic status (Peretti-Watel et al., 2014) are all likely to play a role. While media images of smoking historically have tended to normalise and glamorise smokers (Marron, 2017), current media content often represent smoking as a problem (Chapman and Freeman, 2008; Bain et al., 2017). Age may come into play with regard to whether people have grown up with a ‘normalised’ or a ‘denormalised’ view of smoking as culturally dominating (with the watershed taking place about 1990). Regarding the role of socio-economic status, and whether the associations between economic and cultural capital and the propensity to stigmatise smokers are negative or positive, previous findings vary (Peretti-Watel et al., 2014; Brown-Johnson and Popova, 2016). Following Bourdieu’s theory of class (Bourdieu, 1984; Bernard et al., 2019), however, we may hypothesise that those with the highest economic and cultural capital are more prone to consider smokers a stigmatised group, as these individuals themselves were among the earliest quitters of smoking. Presumably, they are also well aware of smoking having become a declassified low status phenomenon (Sæbø, 2017).

### A cultural approach to perceived public smoker stigma

Drawing on Goffman’s (1990) view of a stigmatised person as discredited and ostracised from society, the stigma concept has been variously approached in studies of health-related behaviour (Yang et al., 2007; Pescosolido and Martin, 2015). While a social-psychological perspective has emphasised stigma as emotional internal processes, prejudicial attitudes and self-stigma, basically as perceived (at the micro level) among the stigmatised (Major and O’Brian, 2005), structural stigma has been outlined by Link and Phelan (2001), highlighting institutional stigma, structural discrimination and the role of power differences. A third cultural perspective has highlighted the role of social context and normative expectations within a society, emphasising that stigma is essentially a moral and intersubjective issue, and arguing that stigmatising conditions illustrate what is at stake for the social actors in a shared social space (Yang et al., 2007; Rao et al., 2008; Kleinmann and Hall-Clifford, 2009).

With inspiration from this latter cultural perspective of stigma, as well as sociologists like Bourdieu (1984) and Archer (1988), in the following we will consider tobacco culture as both product and context of human agency. Both shared and contested meanings of behaviour and deviance are expressed via language and symbols, which, in turn, shape people’s interpretations and social responses to the same behaviours. As stigma is relational in nature, smoker stigma not only resides in individuals or in institutional structures but is also located in the intersubjective space between smokers and non-smokers—in interpersonal actions and communications, as expressions of norms and values. This includes perceptions of *public stigma*—that is, beliefs about stigmatised persons held and communicated by the general public. The concept of public stigma refers both to the perceived level and nature of stigma in overall populations and to the contextual climate of derogatory stereotypes and discrimination regarding certain behaviours (such as smoking) at a certain time and place (Pescosolido and Martin, 2015, pp. 96–101). With smoking having become less cool and glamorous and more marginal and problematic over time (Brandt, 2007), the existence of a public stigma of smokers may be considered as an expression of the historically new ‘unfavourable smoking climate’ described above. The extent to which the public do in fact agree that smokers are stigmatised is also an indicator of the existence a public stigma of smokers.

In Norway and other countries that have reached the final stage of the cigarette epidemic, the current moral standing of the smoker group as such will be dependent on smokers meeting smoking-behaviour obligations and social norms in everyday settings, which again in turn may be influenced by the underlying beliefs and outlooks on life of both smokers (more often from low SES groups) and their non-smoking onlookers (more often from middle or high SES groups). Given the now standard norm of smoke-free environments, public perceptions of smoker stigma may also be part of—or at least associated with—values. But which values, and in what ways? By values, we mean beliefs about basic goals in life, and beliefs about how to proceed to reach these goals. In other words, values refer to goals and means about what is *desirable* (Hitlin and Piliavin, 2004). Theoretically, values are usually considered as derived from social background variables (gender, age, SES etc.). Furthermore, they are more abstract than (but still formative of) specific attitudes, which again tend to govern actual choices and actions (Rokeach, 1973).

Little attention has hitherto been paid to whether (and if so, how) the ‘unfavourable smoking climate’ and the public stigma of smokers associated with tobacco normalisation polices connects with other and more general values among smokers and non-smokers. If the perceived public stigma of smokers turns out to be aligned with, or embedded in, widely shared values, it will be much harder for politicians, health authorities and others to repudiate than if it exists more as an ‘isolated’ and specific attitudinal element of culture.

### Smoking and value oppositions

As the role of values barely has been investigated in sociological tobacco research, there are few published studies to draw from when addressing the significance of values and their possible associations with perceptions of smoker stigma. One exception is a simulation based on the introduction of smokefree restaurants and bars in European countries, which suggests that different patterns of compliance is associated with differences in the normative climate (Dechesne et al., 2013). In a sense, this lack of research focus is surprising, given the strong *cultural* foundation of the idea of smoking denormalisation in tobacco policies. On the other hand, the general literature on values, including what values are, how they should be measured, their causes and consequences etc., is vast, but there is currently little intersubjective consensus on these issues (Hitlin and Piliavin, 2004; Beckers et al., 2012; Dobewall and Rudnev, 2014). Rather than adapting to one of the overall ‘grand’ theoretical perspectives of values as such (for instance those by Inglehart and Welzel, 2005; Schwartz, 2012) at the possible expense of insights into others, we propose instead an exploratory approach, emphasising empirical investigation of four pairs of opposing values. These value oppositions were selected due to their possible relevance (in terms of meaning) to the smoking issue. In the following, we will present these value oppositions, which are previously validated as four out of the 25 ‘lower-order’ value dimensions that are used to construct the two ‘higher-order’ value dimensions ‘modern versus traditional’ and ‘materialist versus idealist’ in the Norwegian Monitor value compass (Hellevik, 1993, 2002). We will also outline how they hypothetically may be linked to the concept of a public stigma of smokers.

#### Puritanism vs. emancipation

The first value opposition contrasts *puritanism* with *emancipation*. Initially used to designate a religious protestant congregation particularly concerned with religious purity in the sixteenth century, puritanism has in modern societies connotatively come to mean any strong (often religiously grounded) moralism that prescribes temperance. As a value, it is thus rooted in the protestant ethics of vocation that Weber (2002) identified as a significant driver in the development of capitalism. In contrast, emancipation as a value refers to becoming free of previous restraints, for instance liberation from religious moralism or traditional ‘non-rational’ mores. This value opposition sits well with ‘traditional’ vs. ‘secular-rational’ values (Inglehart and Welzel, 2005), which is a familiar dimension in previous values research.

Throughout history, a puritan view of smoking has animated much of the political and cultural thinking about tobacco, especially opposition to smoking (Harley, 1993). From this perspective, indulging in tobacco practises is considered a vice that should be avoided. In contemporary public discourse, the label ‘puritan’ has been applied to designate the political will to regulate all types of tobacco as strong as possible, preferably by way of prohibition (Morphett et al., 2020). Thus, holding puritan values may be associated with agreeing that smokers are (and perhaps need to be) stigmatised, as this may be effective in curbing smoking. The value contrast to puritanism is the emancipation from life-restraining temperance ideals and prudent lifestyles, with an emphasis on freedom and a liberating view of doing whatever you want to do, including smoking. Such a view may for instance reflect the historical connection between women’s emancipation from the 1960s onwards and the growth in female smoking, largely a result of the tobacco industry’s deliberate targeting of the autonomous and free female smoker in advertising (Marron, 2017).

#### Conformity vs. individuality

The second value opposition contrasts *conformity* with *individuality*. Conformity as a cultural value involve support of dominant group norms and collective representations, and adherence to accepted practises and standards (such as customs and traditions). Conformity is thus related to hegemonic maintenance of the *status quo*, as in the sociological functionalism of Parsons (1970). In contrast to conformity and its inherent emphasis of normative action, individuality as a cultural value may be a basis for deviance and/or opposition to conformity but may also refer to individual freedom or egoistical considerations and actions. In moral terms, however, individuality refers to the worth of the individual. This value opposition resembles collectivism/individualism, which is another recurring dimension in research on values (Hitlin and Piliavin, 2004).

When it comes to smoking, a requirement to conform to social norms may be associated with a certainty about the existence of a smoker stigma, as tobacco denormalisation policies and attitudes now clearly suggest that smoking is unacceptable (Chapman and Freeman, 2008). Consequently, a contemporary ‘conformist’ who considers prevailing laws and norms to be directive for behaviours and outlooks is not only likely to think that smokers are stigmatised, but perhaps even rightfully so (just like the puritans). This stands in contrast to the 1970s and 80s, when conformism was more likely to reflect that smoking was normal and even cool, especially among young people (Stewart-Knox et al., 2005). The value contrast to conformity is individuality: opposing any submission to norms that a person perhaps disagrees with or does not think should be a behavioural norm at all—including the paternalist idea of denormalising smoking (Dennis, 2011).

#### Tolerance vs. intolerance

The third value opposition contrasts *tolerance* with *intolerance*. Tolerance as a value involves acceptance of actions, utterances, lifestyles or individuals one dislikes or disagrees with. It also involves recognising the rights of others, not only to think differently than oneself or what the majority think, but also to live in accordance with their opinions, e.g., due to religious or political reasons. As such, tolerance is a value orientation directed toward difference (Hjerm et al., 2020), firmly rooted in political liberalism (Mill, 1974), ethics (Rawls, 1999) and human rights, and also related to the value of universalism. Intolerance on the other hand, refers to lack of tolerance, lenience, or open-mindedness toward different opinions than one’s own (Verkuyten and Kollar, 2021). This value opposition resembles the ‘self-expression/post-materialism’ vs. ‘survival/materialism’ dimension in the works of Inglehart and Welzel (2005).

As long as cigarettes are legal products and smoking is a legal practise, a cultural ideal of tolerance may imply that smoking and smokers should be accepted as is, without the state seeking to disgrace citizens who engage in such practises. As smoking over time has been denormalised and marginalised, the approach of the in-group of non-smokers to the out-group of smokers may vary, however, from a tolerating acceptance of a preference to smoke on the one hand (including in legislation, see Muggli et al., 2010) to an intolerant and moralising condemnation on the other (Rozin and Singh, 1999; Moore, 2005). Thus, holding tolerant views may be associated with a tendency to disagree with devaluating statements such as ‘most people think less of people who smoke.’ Holding intolerant views may be associated with a tendency to agree with such statements.

#### Status vs. anti-status

The fourth value opposition contrasts *status* with *anti-status*. In sociology, status refer to the rank, wealth and power deriving from the individuals’ placements in hierarchical social positions. Status is rooted in economic capital that again may be converted into social and cultural capital due the logic of lifestyle distinction in social space (Bourdieu, 1984). As a value, status refers to acknowledging symbolic conspicuous consumption and ‘abstract goods’ like prestige, reputation and respect as legitimate means to gain influence in social interaction and games of power. In contrast, anti-status as a value refers to rejecting the significance of such symbolic power rules in ‘status plays,’ as they for instance may be considered as superficial, shallow or rigid, or as illegitimate means to control people and resources. Anti-status suggests that other things than material wealth may have value in life, such as authenticity, love, or the wonders of nature. While holding a ‘status’ value tends to score high on materialism, ‘anti-status’ often scores higher on idealism (Hellevik, 1993), and thus also on post-materialism.

Regarding status and prestige, cigarettes and smoking have historically moved from being a product and practise that radiated status and being cool to the polar opposite; in a political-cultural context of tobacco denormalisation, regular daily smoking tends to radiate addiction, lack of control and being an uncool ‘loser’ (Sæbø, 2017). Emphasising status as a value (i.e., wanting to make an impression on others, wishing to gain the respect and admiration of others) may therefore be associated with a denormalised view of smoking, an acknowledgment of smoking as being uncool and having a low status and, consequently, a tendency to agree that, in general, smokers are stigmatised. Being anti-status value-wise, on the other hand, may involve opposition to a view of letting others define what is worthwhile or perhaps refuting ‘external conditions’ as a guide to judgements of what is impressive in life, and consequently, also disagreeing with smokers being stigmatised.

### Research problem

Little is known about the dissemination of stigmatising attitudes toward people who smoke at the national level, including their associations with personal values. In this paper, we explore the existence of a perceived public stigma in Norway and its association with the four value oppositions laid out above as well as other possible predictors. More precisely, we aim to identify (a) the spread of perceived public smoker stigma in the Norwegian population, and (b) whether the tendency to agree with a perceived public smoker stigma differs by expressed value opposites after controls for demographics, socio-economic status and smoking behaviour, including cessation plans among current smokers.

## Data and methods

### Data

Two data sets from the biennial cross-sectional and nationally representative *Norwegian Monitor* survey were employed: *Norwegian Monitor 2011* (*N* = 3,980, N smokers = 934, N ex-smokers = 1,242) and *Norwegian Monitor 2013* (*N* = 3,812, N smokers = 762, N ex-smokers = 1,211). This survey is organised by the public opinion agency IPSOS/Synnovate in collaboration with the University of Oslo. For the purposes of the analyses in this paper, and to achieve sufficient statistical power to address the relatively small sub-groups of various smoking behaviours, these data sets were merged (total *N* = 7,792, total N smokers = 1,696, total N ex-smokers = 2,265).

Probability sampling based on telephone records was used to draw a random sample of the adult Norwegian population (>15 years). The exact size of the gross sample was not known due to uncertainty about the status of the telephone numbers (i.e., whether they were active or not). Respondents who agreed to participate in a short introductory telephone interview (2011, *N* = 10,248; 2013, *N* = 10,098) were then invited to participate in the main self-administered part of the survey, thereby constituting the eligible sample. Those who responded to the questionnaire constitute the analytical sample of the current study.

Of those respondents who made up the eligible sample, 39% answered their questionnaires in 2011 while the return rate fell to 37% in 2013 (Hellevik, 2015). There was a lower response rate for males and young age groups, and a higher response rate from respondents with long education. Nevertheless, a collation of the final sample with the official statistics on smoking rates from Statistics Norway displays a high degree of correspondence (Hellevik, 2015).

The IPSOS group responsible for the survey has committed to international compliance with data protection law and ESOMAR ethical guidelines. Informed consent was obtained, and the data was anonymised before being submitted to the authors. Thus, indirect identification of respondents is not possible.

### Measures

*Perceived public smoker stigma* was measured through the following question: ‘How much do you agree or disagree with the following statement? Most people think less of a person who smokes.’ The response categories were: ‘totally agree,’ ‘partially agree,’ ‘partially disagree’ and ‘totally disagree.’ This indicator of public smoker stigma was borrowed from the devaluation index of Stuber et al. (2009, p. 592). Responses were recoded into ‘totally or partially agree’ and ‘totally or partially disagree.’

To assess *smoking status*, respondents were asked: ‘Which of the following statements best describes your situation?’ The response categories were: ‘I smoke and have no plans to quit in the next 6 months’ (=1), ‘I smoke but am seriously considering quitting in the next 6 months’ (=2), ‘I smoke and have decided to quit in the next 30 days’ (=3), ‘I used to smoke, but quit less than 6 months ago’ (=4), ‘I used to smoke, but quit more than 6 months ago’ (=5), ‘I used to smoke, but quit more than 5 years ago’ (=6), and ‘I have never smoked’ (=7).^1^ This question was recoded into ‘current smoker without any plans to quit’ (response 1), ‘current smoker with plans to quit’ (2,3), ‘recent quitter—i.e., quit less than 5 years ago’ (4,5), ‘long-term quitter - i.e. quit more than 5 years ago’ (6), and ‘never-smoker’ (response 7). All respondents were also asked whether they *live with others who smoke* (no/yes).

Regarding *value oppositions*, four theoretically relevant additive value indexes as defined by the *Norwegian Monitor* value compass (Hellevik, 1993, 2002), were utilised as independent variables. These were:

- *Puritanism/emancipation*. This index is based on the following questions: ‘Sexual experiences before marriage helps make the marriage happier’ (‘totally agree,’ ‘partially agree,’ ‘impossible to answer,’ ‘partially disagree,’ ‘totally disagree’) and ‘Do you think so-called pornographic magazines, books and writing should be banned, or do you think such items should be sold freely?’ (‘should be banned,’ ‘should be freely available,’ ‘not sure’).
- *Conformity/individuality*. This index is based on the following questions: ‘How important do you think it is that on special occasions you dress and act according to custom and practise?’ (‘very important,’ ‘quite important,’ ‘less important,’ ‘does not matter’), and ‘The worst thing I know is people who cannot be like most people’ (‘totally agree,’ ‘partially agree,’ ‘impossible to answer,’ ‘partially disagree,’ ‘totally disagree’).
- *Tolerance/intolerance*. This index is based on the following questions: ‘There are many opinions that should never be expressed on radio and television,’ ‘People should be able to look, dress and live as they like, whether or not others like it,’ and ‘It should be reasonable to expect that foreigners who come to settle in Norway live like Norwegians’ (response categories on all three questions: ‘totally agree,’ ‘partially agree,’ ‘partially disagree,’ ‘totally disagree,’ ‘impossible to answer’).
- *Status/anti-status*. This index is based on the following questions: ‘I try to acquire things that make an impression on others,’ ‘Nice house, expensive car and fancy clothes engender the admiration of others (both ‘totally agree,’ ‘partially agree,’ ‘partially disagree,’ ‘totally disagree,’ ‘impossible to answer’) and ‘If you had one wish today, which of the following alternatives would you choose?’ (Would choose this alternative first: ‘To be even more respected by people with whom I associate’).

All indexes were thus based on questions with different wordings, to control for possible ‘yes-saying.’ The utilised variables were first recoded, with ‘impossible to answer’ and ‘not sure’ categories being placed in the middle, and then merged into an additive index, with the outer categories representing opposite value ‘poles’ (Hellevik, 2002). Each respondent was accordingly placed on an additive scale, ranging from 9 to 12 when it comes to our four value indexes. For the analytical purposes of this paper, these value indexes were normalised to vary between 0 and 100 (in respect of the descriptive statistics) and recoded into quartiles (in respect of the regression model).^2^

Regarding background variables, we controlled for SES (the highest level of completed education: ‘primary school,’ ‘lower and upper secondary school,’ ‘university, low grade,’ and ‘university, high grade,’ and household income: ‘<NOK 499,000,’ ‘NOK 500–799,000,’ ‘NOK 800–999,000,’ and ‘+NOK 1 million’), gender and age group (‘15–24,’ ‘25–39,’ ‘40–59,’ ‘60+’).

### Statistical analyses

To address the extent and dissemination of perceived public stigma, a descriptive statistical analysis was applied. To specifically assess the central tendency of the value indexes, a normalised distribution was applied. To address differences between groups, binary logistic regression was utilised. The latter analysis was conducted on two levels. First, the unadjusted associations between all the independent variables and public stigma were explored. Then, multiple controls were performed, using a block-wise approach, to identify any possible mediating role of values. Here, the tendency to agree with a perceived public smoker stigma was regressed on socio-demographic variables (gender, age, education, income) in the first step, socio-demographics and smoking in the second step, socio-demographics and value opposites in the third step, and socio-demographics, value opposites and smoking status in the fourth and last step. To report model goodness of fit and explained variance, Hosmer-Lemeshow and Nagelkerke tests were performed. All results from the logistic regression analysis were based on respondents who had answered all the questions.

Multicollinearity among the value indexes was investigated but was not found to be a statistical problem (see correlation matrix in Table 1).

**Table 1:** Correlation matrix for the value indexes (before recoding into quartiles).

	VI1 (puritanism/emancipation)	VI2 (conformity/individuality)	VI3 (tolerance/intolerance)
VI2 (conformity/individuality)	0.002 (0.829)	–	
VI3 (tolerance/intolerance)	0.146 (<0.001)	−0.349 (<0.001)	–
VI4 (status/anti-status)	−0.132 (<0.001)	−0.122 (<0.001)	0.111 (<0.001)

Pearson’s R (significance level in parenthesis). *N* = 7,792.

The analyses were conducted using SPSS v28.

## Results

### Descriptives

The descriptive distributions of the variables employed are laid out in Table 2.

**Table 2:** Descriptive statistics.

Smoker stigma	Partially or fully disagree	38.6
	Partially or fully agree	59.1
	No info	2.3
Gender	Male	46.2
	Female	53.8
Age	15–24	12.8
	25–39	16.6
	40–59	38.9
	60+	31.7
Education	Primary	10.7
	Secondary	30.7
	University, low grade	34.6
	University, high grade	23.8
	No info	0.2
Income, household	<NOK 499,000	30.5
	500–799	29
	800–999	16.7
	+NOK 1 million	19.6
	No info	4.1
Smoking status	Never-smoker	46.1
	Quit >5 years ago	24.8
	Quit <5 years ago	6.7
	Smoker with plans to quit	9.9
	Smoker with no plans to quit	8.2
	No info	4.3
Live with others who smoke	No	82.9
	Yes	14.8
	No info	2.3
Puritanism/emancipation (value index I)	Q1 (puritanism)	24.8
	Q2	29.8
	Q3	15.6
	Q4 (emancipation)	29.9
	*Means*	*61,8*
Conformity/individuality (value index II)	Q1 (conformity)	20.4
	Q2	15.7
	Q3	30.7
	Q4 (individuality)	33.1
	*Means*	*47.2*
Tolerance/intolerance (value index III)	Q1 (tolerance)	20.4
	Q2	26.7
	Q3	25.7
	Q4 (intolerance)	27.1
	*Means*	*39.3*
Status/anti-status (value index IV)	Q1 (status)	15.3
	Q2	42.9
	Q3	10.2
	Q4 (anti-status)	31.6
	*Means*	*72*

Percentages, *N* = 7,792.

59.1% of the total sample agree with the statement that ‘most people think less of a person who smokes,’ rising to 67.7% among daily smokers (table not shown). The tendency to agree that smokers are looked down upon is also shared by a majority of the never-smokers (57.8%, table not shown).

There were 46.1% never-smokers in the sample, 31.5% ex-smokers and 18.1% current smokers. A slight majority of the current smokers express plans to quit.

The mean scores for the value indexes were 61.8% for the ‘puritanism/emancipation’ index, 47.2% for the ‘conformity/individuality’ index and 39.3% for the ‘tolerance/intolerance’ index. Finally, for the ‘status/anti-status’ index the mean score is 72.0%, suggesting a strong inclination in the sample to express an ‘anti-status’ rather than ‘status’ value position.

The strongest inter-correlation between the value indexes was found between ‘tolerance/intolerance’ and ‘conformity/individuality’ (Pearson’s *R* = −0.349, *p* < 0.001), which suggests that ‘tolerance’ tend to go with ‘individuality’ and ‘intolerance’ with ‘conformity.’ There were statistically significant, but less notable, associations between all the value indexes, bar one (see Table 1).

### Regressing public smoker stigma: unadjusted associations

The logistic regression analysis is presented in Table 3. Unadjusted significant associations were found for all variables utilised, except for the ‘tolerance/intolerance’ value index and ‘living with other smokers.’

**Table 3:** Logistic regression.

Variables	Unadjusted ORs	Adjusted ORs (block-wise modelling)				Model 1: Socio-demographics (A)	Model 2: Socio-demographics (A) + smoking behaviour (B)	Model 3:Socio-demographics (A) + value opposites (C)	Model 4: Socio-demographics (A) + smoking behaviour (B) + value opposites (C)
Gender					
-Male (ref. cat.)	1.00	1.00	1.00	1.00	1.00
-Female	1.14**	1.14*	1.13*	1.26***	1.24***
Age					
−15–24 (ref. cat.)	1.00	1.00	1.00	1.00	1.00
–25–39	0.96	0.86	0.75**	0.91	0.80*
–40–59	0.77**	0.68***	0.57***	0.78**	0.65***
–60+	0.74***	0.73***	0.63***	0.86	0.74**
Education					
-Primary (ref. cat.)	1.00	1.00	1.00	1.00	1.00
-Secondary	0.92	0.94	0.92	0.93	0.92
-University, low grade	1.07	1.08	1.11	1.1	1.12
-University, high grade	1.23*	1.18	1.24*	1.21	1.27*
Income, household					
-<499,000 (ref. cat.)	1.00	1.00	1.00	1.00	1.00
–500–799	0.94	0.96	1.00	0.96	1.00
–800–999	1.01	1.02	1.07	1.01	1.06
−1 million+	1.53***	1.50***	1.61***	1.42***	1.52***
Smoking status					
-Never-smoker (ref. cat.)	1.00		1.00		1.00
-Quit >5 years ago	1.08		1.25**		1.22**
-Quit <5 years ago	1.50***		1.62***		1.58***
-Smoker with quit plans	1.90***		2.42***		2.34***
-Smoker, no quit plans	1.29**		1.62***		1.57***
Live with others who smoke					
-No (ref. cat.)	1.00		1.00		1.00
-Yes	0.91		0.79**		0.77***
Puritanism/emancipation					
-q1 (ref. cat.)	1.00			1.00	1.00
-q2	1.24**			1.23**	1.17*
-q3	1.25**			1.21*	1.16
-q4	1.53***			1.48***	1.39***
Conformity/individuality					
-q1 (ref. cat.)	1.00			1.00	1.00
-q2	1.04			1.02	1.02
-q3	0.98			0.95	0.96
-q4	0.86*			0.86*	0.86
Tolerance/intolerance					
-q1 (ref. cat.)	1.00			1.00	1.00
-q2	1.03			1.03	1.03
-q3	0.97			0.96	0.96
-q4	1.00			1.06	1.07
Status/anti-status					
-q1 (ref. cat.)	1.00			1.00	1.00
-q2	0.79**			0.86*	0.85*
-q3	0.84			0.88	0.88
-q4	0.56***			0.63***	0.63***
Hosmer-Lemeshow		0.260	0.211	0.024	0.526
Nagelkerke		0.018	0.039	0.036	0.054

Dependent variable: agree with the statement ‘most people think less of a person who smokes.’ Unadjusted and adjusted odd ratios (OR). *N* = 6,921. *p < 0.05, **p < 0.01, ***p < 0.001.

### Adjusted associations between background variables and public smoker stigma

Adjusting for other variables in the block-wise regression, the effects of gender (women more in agreement), age (15–24 years most in agreement) and income (+NOK 1 million most in agreement) were maintained in all the 4 models tested. The unadjusted effect of education disappeared after multiple controls for other socio-demographical variables (model 1) and value opposites (model 3) but reappeared in model 2 and the full model (after the inclusion of smoking status).

### Adjusted associations between smoking status and perceived public smoker stigma

‘Smokers with plans to quit’ is the smoking status sub-group that expresses the strongest tendency to agree with a perceived public smoker stigma, controlling for all other factors (OR = 2.34 in the full model 4). Also, those ex-smokers who quit recently are more often in agreement with the assertion that smokers are stigmatised (OR = 1.58 in model 4) than ex-smokers who quit more than 5 years ago (OR = 1.22 in model 4). (A supplementary test using ‘quit >5 years ago’ as a reference category suggests that this difference is significant). Living with others who smoke is also associated with lower agreement with public stigma (OR = 0.77) in the full model.

Note that the OR for agreeing with a perceived stigma among current smokers with plans to quit increases from 1.90 unadjusted to 2.34 in the fully adjusted model. This latter finding is most likely due to strong confounding with ‘living with others who smoke.’

### Adjusted associations between value opposites and perceived public smoker stigma

The four value indexes have no common resemblance to perceived public smoker stigma. However, the associations with the value indexes of puritanism/emancipation and status/anti-status remain significant in the adjusted models. A higher score on the puritanism/emancipation index is associated with higher odds for agreeing with smoker stigma (i.e., emancipates more widely agree with smoker stigma, while puritans agree to a lesser extent). A lower score on the index for status/anti-status values, on the other hand, is associated with higher ORs for agreeing with public smoker stigma, i.e., those who are concerned with status are more likely to agree with the stigma of smokers compared to those holding an anti-status value. The small, significant association between the conformity/individuality index and the outcome variable perceived public smoker stigma disappears after controlling for smoking status.

There was no evidence that values play any mediating role in our modelling.

## Discussion

### Main findings

The tendency to agree with the statement ‘most people think less of a person who smokes’ is quite high in the Norwegian population, including among never-smokers, and certainly higher than what was found in a recent French study (Peretti-Watel et al., 2014). People who agree with a perceived public smoker stigma are more likely to be young, female, with a high level of education and income, former or current smokers, and espoused to the values of emancipation and status. Inasmuch as the overall tendency to agree with a perceived public smoker stigma is as high as it is, this opinion is a shared cultural characteristic in Norway. This is noteworthy, as the aim of the policy is to denormalise the act of smoking, not stigmatise people who smoke.

### Interpretation of findings as regards value opposites

To overcome the neglect of the role of values in sociological tobacco research in general, and in smoker stigma research in particular, we have in this article suggested an exploratory approach, to test whether four value oppositions with hypothetical relevance to the smoking issue were associated with the stigmatisation of smokers. Our approach is based on the premise that values provide a direction toward what is worth striving for and what is considered important in society; hence, they may also serve as motivational underpinnings of attitudes and perceptions of smokers and smoking behaviour (Hitlin and Piliavin, 2004). However, the indexes for intolerance/tolerance and individuality/conformity were not found to be significantly associated with public smoker stigma in our study after multiple controls. Thus, the perceived public stigma of smokers may appear to belong to other cultural dimensions than these. One should perhaps expect that tolerant people would show a higher acceptance of a norm-deviant behaviour like smoking, but this does not seem to be the case from our data. Or people may support a general ideal of tolerance, but still evaluate smokers and smoking negatively or positively, depending perhaps on their personal experience of smoking behaviour and/or how they view addictive behaviour in general. It should also be noted that our dependent variable primarily measures the extent to which people agree that smokers are usually looked down upon (or ‘devalued’). We have not studied whether or not this is considered ‘right’ or whether respondents think that smokers ‘deserve’ to be looked down upon.

What we did find is that respondents favouring puritan values over emancipation values were less likely to suggest that smokers are stigmatised, while those who expressed an emphasis on status values were more inclined to agree with the public stigma of smokers than those who were anti-status. Initially, we expected that puritans were more likely to agree that smokers were stigmatised, but this turned out not to be the case. Puritanism implies expectancy of moral discipline, but even if puritans are likely to be against smoking and pro-denormalisation, they may perhaps be less sensitive to the explicit issue of stigma. Those who support emancipation on the other hand, may be more aware of the unintended consequences of strong regulation and denormalisation of smokers. While, in the case of status values, if status affects you, you are concerned about how other people look at you (Bourdieu, 1984). When opting to move upwards in social hierarchies, you may want to look down on others, not least those positioned socially close to you who do not pay adequate attention to distinctive status behaviour and consumption (such as smokers).

The statistically significant role of status and emancipation values may also reflect that people’s notions of smokers are characterised by assumptions of smokers as addicted persons. A dependency on substances such as nicotine and alcohol indicates that the persons are not in complete control, that they are not free, but governed by outside forces. In contemporary cultures and work environments that cherish qualities such as adaptability and flexibility, expressing a lack of control and absence of self-regulation is likely to be regarded as a problem (Gilbert et al., 1998). And while the use of tobacco and intoxicants is often perceived as cool by young people, not even young people think that users who lose control and become addicted are cool (Scheffels and Schou, 2007). Those who favour emancipation and status values are likely to rank autonomy and freedom from dependence highly, and these are qualities that they may think addicted smokers lack.

Furthermore, it is interesting to note that those respondents with the highest economic (income) and cultural (education) capital express higher agreement with the public stigma of people who smoke than those respondents with lower levels of capital. This may suggest a possible paternalistic concern for smokers, but also be associated with distinction (Bourdieu, 1984), as those privileged in terms of capital may distance themselves from smoking as a low status phenomenon and also the notion of ‘most people’ referred to in the question that measures perceived public smoker stigma. This finding may also reflect the occurrence of a partly elitist and ‘degrading’ view of smokers among high SES groups, including many public health representatives (Lupton, 1995; Dennis, 2011).

Taken together, the present findings indicate that the perceived public stigma of smokers in Norway is most common among high-status groups, and those who emphasise status (and emancipation) as values. As such, our findings are congruent with the Stuber group’s findings from New York City (Stuber et al., 2008, 2009).

When it comes to the effects of age, adolescents, and young adults (<25 years) are more in agreement with a public stigma of smokers than older respondents. In contrast to the youngest, who have been raised with a denormalised view of smoking, older respondents grew up in a period when smoking was considered as normal (and even glamorous), so it may fit with a generational age explanation that older respondents to a lesser extent tend to agree with the public smoker stigma.

### Never-smokers and smokers views on the perceived public stigma of smokers

In keeping with our cultural approach to smoker stigma, we have emphasised that the perceived public stigma of smokers is intersubjective and relational (Yang et al., 2007). It does not primarily reside within individuals or in power structures; it is played out in terms of what is at stake, in our case, in negative beliefs about persons who smoke, as these are held and communicated by the general public. Our public stigma measure is thus an operationalisation of what is basically a triangular model, consisting of individual respondents who express their perception of how the relationship between the majority (most people) and people who smoke is today. We have interpreted this measure as an expression of the mood that prevails around the moral status of today’s smokers.

However, never-smokers and smokers view the question of smoker stigma from different positions and with different perspectives. For never-smokers, the perceived public stigma of people who smoke is basically a theoretical construct regarding ‘others’ who are quite likely to be socially distanced from themselves—compare the tendency to social marginalisation of smokers (Graham, 2012) and the existence of smoking enclaves among current smokers (Thompson et al., 2007). An exception to this is those who live with others who smoke (Peretti-Watel et al., 2014). This social distance may involve a greater interpretational variety of smoking as a cultural phenomenon than among smokers, for whom expectations of being smoke-free may have been internalised and embodied in quite a uniform fashion. For people who smoke, the perceived public stigma of smoking is very real and is associated with a habit that is difficult to refrain from and which may have several potentially damaging consequences, such as health hazards and disapproving looks from relatives and onlookers (McCool et al., 2013). The development of new nicotine-delivering devices such as e-cigarettes may possibly ‘renormalise’ smoking to some degree, but vaping practises may also inherit the stigma associated with cigarette smoking (Tokle and Pedersen, 2019).

As expected, people who smoke agreed the most with the public smoker stigma, in line with previous research in which 80% of smokers reported that society disapproves of smoking (Hammond et al., 2006). In the total sample, ‘smokers with current plans to quit’ is the smoking status group that expresses the strongest tendency to agree with the perceived public smoker stigma. Smokers who plan to quit (or who recently quit) use several motivational techniques to stay abstinent or to achieve successful cessation. Among motives for quitting reported by smokers are the negative image that smoking has in society, a motive more often expressed by more highly educated smokers (Baha and Le Faou, 2010). In cultures where healthy behaviour and being tobacco-free are widespread social norms, daily smoking may signal a lack of control, psychological reactance, and recklessness (Lupton, 1995; Sæbø, 2017). Even if the perceived public stigma of smokers among people who smoke should not be commingled with self-stigma (Bracke et al., 2019), a social identity as a ‘smoker’ may obviously be troublesome in cultural contexts where smoking is considered as unacceptable deviance. Nevertheless, in contrast to stigma associated with other health issues (such as HIV/AIDS and chronic mental health ailments), the smoker stigma may be overcome and disappear if smoking cessation proves to be successful (Bayer, 2008). Because stigma (at least in theoretical terms) is a potential stimulus or incentive to smoking cessation, there might have been a higher acceptance of stigma as the ‘price to pay’ in tobacco control than in other fields of health governance (Bayer and Stuber, 2006). However, the causal direction between intention/motivation to quit and perceived stigma among smokers is not known, nor can it be adequately addressed in our study because of the cross-sectional perspective.

Finally, a possible interesting ‘policy implication’ of our study should be mentioned. The finding that the perceived public smoker stigma does not seem to be compellingly associated with underlying values (at least as we measured them in the present study) suggests that smoker stigma may be easier for policy makers and health governors to counter than if it had been a greater part of (or more strongly embedded in) values. Future studies should investigate whether other measurements of personal and cultural values—e.g., the operationalisations of Inglehart and Welzel (2005), or Schwartz (2012)—provide any other type of findings that maintain or changes this interpretation of the current findings.

### Limitations

There are some important limitations to our study.

First, the applied stigma measure is quite a crude operationalisation of stigma, which is essentially a multidimensional phenomenon (Link and Phelan, 2001; Pescosolido and Martin, 2015). It measures agreement with one aspect of stigma only, although admittedly an important aspect, namely, the tendency to agree that most people look down on, and thus stigmatise, a person who smokes. This is what the literature calls ‘public stigma.’ It is a measure of what people believe to be the social norm, not necessarily their personal opinion. Importantly, it measures agreement with a descriptive statement only; it does not refer to any acceptance of stigma. This methodological issue may also help explain the missing concordance between some of the values addressed in this study and the stigma of smoking. Future studies using a broader measurement of the stigma complex are needed to address this issue.

Second, the value indexes applied are based on original questions that perhaps may appear a little outdated today. The value indexes are also generic, with wording that does not explicitly relate to smoking. However, these indexes have been applied as components of underlying value dimensions in Norwegian value research since 1985 and are therefore well validated (Hellevik, 1993, 2002). Empirically, they do actually distinguish between people’s views and have done so for more than three decades. Still, in the future it may be useful to also explore whether explicit smoking norms possibly acts as intermediaries between cultural values and the perceived public stigma of smokers and whether this mechanism differs between different national cultures (Dechesne et al., 2013).

Third, the data is based on self-reporting, which is always a limitation when it comes to measuring the extension or spread of phenomena (such as public smoker stigma). However, as our dependent variable is actually an attitude or opinion, the self-reporting is probably less of a problem than if the variable had been a behaviour.

Finally, in the sample, highly educated respondents are somewhat overrepresented. As those with low education (many of whom are still smokers) are underrepresented in this sample, we may have underestimated both the level of smoking behaviour and the social inequalities in smoking behaviour underlying the perception of public stigma.

## Conclusion

Smoking denormalisation policies have sought to obtain public health goals (lower smoking prevalence and less tobacco-related death and disease) through cultural redefinition of the meaning of smoking. Although this strategy seems to have achieved its missions in many countries, the redefinition of smoking from ‘normal’ to ‘unacceptable’ has also had the unfortunate and unintended consequence of contributing to stigmatise the remaining smokers, especially smokers who want to quit but who have not yet succeeded with this task. The perceived stigma of smokers among the public is one (infrequently utilised) way to measure the extent of this stigmatisation. In this article, we have identified factors that predict agreement (or disagreement) with a public stigma of smokers, with a particular emphasis on the role of general value opposites. Our empirical results indicate that the perceived public stigma of smokers is high in Norway and that it varies with two of the four indexes of value opposites assessed (‘puritanism/emancipation’ and ‘status/anti-status’) as well as with socio-demographics. The findings also corroborate the existence of a statistically independent cleavage between smokers and non-smokers in the perceived public stigma of smokers, an issue that in itself is culturally charged.

## Data availability statement

The original contributions presented in the study are included in the article/supplementary material, further inquiries can be directed to the corresponding author.

## Ethics statement

The studies involving humans were approved by the Norwegian Data Protection Authority. The studies were conducted in accordance with the local legislation and institutional requirements. Written informed consent for participation in this study was provided by the participants’ legal guardians/next of kin.

## Author contributions

All authors listed have made a substantial, direct, and intellectual contribution to the work and approved it for publication.

## Footnotes

^1^In 2013, a technical filter glitch on the part of IPSOS/Synnovate resulted in around 50% missing responses among non-smokers on this variable. There was no systematic pattern but rather a coincidence about who had answered the question or not, by gender, age, and region. The missing responses were therefore assigned a random response from a matrix of existing data on age (to account for the higher likelihood of having smoked previously by age) in the column and actual responses of those who had answered in the row. Thus, the response probability of those who had answered was used to allocate assigned answers to those who had not answered (‘interpolation’). Additional tests were performed on all analyses presented in this article, by 2011 and 2013. The results did not suggest that the interpolations created artificial or deviating results. To achieve statistical power in our analysis, it was therefore decided to pool the two data sets.

^2^As shown in Table 2, this recoding resulted in a somewhat skewed distribution of quartiles for three of the four value indexes. Additional tests were thus performed, entering these indexes as linear variables instead of categorical variables in the logistic regression models. Results did not differ substantially (that is, provide other significant associations) from what is reported in Table 3.

## References

[ref1] Archer MS (1988) Culture and agency: the place of culture in social theory. Cambridge: Cambridge University Press.

[ref2] Baha M. Le Faou A. L. (2010). Smokers’ reasons for quitting in an anti-smoking social context. Public Health 124, 225–231. doi: 10.1016/j.puhe.2010.02.011 20371089

[ref3] Bain J. Weishaar H. Semple S. Duffy S. Hilton S. (2017). Vulnerable children, stigmatised smokers: the social construction of target audiences in media debates on policies regulating smoking in vehicles. Health 21, 633–649. doi: 10.1177/1363459316633279, PMID: 27457688 PMC5639949

[ref4] Bayer R. (2008). Stigma and the ethics of public health: not can we but should we. Soc. Sci. Med. 67, 463–472. doi: 10.1016/j.socscimed.2008.03.017 18502551

[ref5] Bayer R. Stuber J. (2006). Tobacco control, stigma, and public health: rethinking the relations. Am. J. Public Health 96, 47–50. doi: 10.2105/AJPH.2005.071886, PMID: 16317199 PMC1470446

[ref6] Beckers T. Siegers P. Kuntz A. (2012). Congruence and performance of value concepts in social research. Surv Res Methods 6, 13–24. doi: 10.18148/srm/2012.v6i1.5093

[ref7] Bell K. McCullough L. Salmon A. Bell J. (2010). ‘Every space is claimed’: smokers’ experiences of tobacco denormalisation. Sociol. Health Illn. 32, 914–929. doi: 10.1111/j.1467-9566.2010.01251.x, PMID: 20525013

[ref8] Bernard M. Fankhänel T. Riedel-Heller S. G. Luck-Sikorski C. (2019). Does weight-related stigmatisation and discrimination depend on educational attainment and level of income? A systematic review. BMJ Open 9:e027673. doi: 10.1136/bmjopen-2018-027673, PMID: 31740462 PMC6886928

[ref9] Bourdieu P. (1984) Distinction: a social critique of the judgement of taste. London: Routledge & Kegan Paul.

[ref10] Bracke P. Delaruelle K. Verhaeghe M. (2019). Dominant cultural and personal stigma beliefs and the utilization of mental health services: a cross-national comparison. Front. Sociol. 4:40. doi: 10.3389/fsoc.2019.00040 33869363 PMC8022809

[ref11] Brandt A. M. (2007) The cigarette century: the rise, fall and the deadly persistence of the product that defined America. New York, NY: Basic Books.

[ref12] Brown-Johnson C. G. Popova L. (2016). Exploring smoking stigma, alternative tobacco product use, and quit attempts. Health Behav Policy Rev 3, 13–20. doi: 10.14485/HBPR.3.1.2, PMID: 27088103 PMC4829360

[ref13] Chapman S. Freeman B. (2008). Markers of the denormalisation of smoking and the tobacco industry. Tob. Control. 17, 25–31. doi: 10.1136/tc.2007.021386, PMID: 18218803

[ref14] Dechesne F. Di Tosto G. Dignum V. Dignum F. (2013). No smoking here: values, norms and culture in multi-agent systems. Artif. Intell. Law 21, 79–107. doi: 10.1007/s10506-012-9128-5

[ref15] Dennis S. (2011). Smoking causes creative responses: on state antismoking policy and resilient habits. Crit. Public Health 21, 25–35. doi: 10.1080/09581596.2010.529420

[ref16] Dobewall H. Rudnev M. (2014). Common and unique features of Schwartz’s and Inglehart’s value theories at the country and individual levels. Cross-Cult. Res. 48, 45–77. doi: 10.1177/1069397113493584

[ref17] Evans D. N. Furst R. T. (2016). Stigma and outdoor smoking breaks: self-perceptions of outdoor smokers in Manhattan. Soc. Theory Health 14, 275–292. doi: 10.1057/sth.2015.33

[ref18] Evans-Polce R. J. Castaldelli-Maia J. M. Schomerus G. Evans-Lacko S. E. (2015). The downside of tobacco control? Smoking and self-stigma: a systematic review. Soc. Sci. Med. 145, 26–34. doi: 10.1016/j.socscimed.2015.09.026 26439764 PMC4630105

[ref19] Gibson B. (1998). Nonsmokers’ attributions for the outcomes of smokers: some potential consequences of the stigmatization of smokers. J. Appl. Soc. Psychol. 28, 581–594. doi: 10.1111/j.1559-1816.1998.tb01721.x

[ref20] Gilbert G. R. Hannan E. L. Lowe K. B. (1998). Is smoking stigma clouding the objectivity of employee performance appraisal? Public Pers. Manag. 27, 285–300. doi: 10.1177/009102609802700301

[ref21] Goffman E. (1990) Stigma: notes on the management of spoiled identity. Harmondsworth: Penguin Books.

[ref22] Graham H. (2012). Smoking, stigma and social class. J. Soc. Policy 41, 83–99. doi: 10.1017/S004727941100033X

[ref23] Hammond D. Fong G. T. Zanna M. P. Thrasher J. F. Borland R. (2006). Tobacco denormalization and industry beliefs among smokers from four countries. Am. J. Prev. Med. 31, 225–232. doi: 10.1016/j.amepre.2006.04.004, PMID: 16905033

[ref24] Harley D. (1993). The beginnings of the tobacco controversy: puritanism, James I, and the royal physicians. Bull. Hist. Med. 67, 28–50. PMID: 8461637

[ref25] Hefler M. Carter S. M. (2019). Smoking to fit a stigmatised identity? A qualitative study of marginalised young people in Australia. Health 23, 306–324. doi: 10.1177/1363459317745690 29188726

[ref26] Hellevik O. (1993). Postmaterialism as a dimension of cultural change. Int J Public Opin 5, 211–233. doi: 10.1093/ijpor/5.3.211

[ref27] Hellevik O. (2002). Age differences in value orientation – life cycle or cohort effects? Int. J. Public Opin. Res. 14, 286–302. doi: 10.1093/ijpor/14.3.286

[ref28] Hellevik O. (2015). Extreme nonresponse and response bias. Qual. Quant. 50, 1969–1991. doi: 10.1007/s11135-015-0246-5

[ref29] Hitlin S. Piliavin J. A. (2004). Values: reviving a dormant concept. Annu. Rev. Sociol. 30, 359–393. doi: 10.1146/annurev.soc.30.012703.110640

[ref30] Hjerm M. Eger M. A. Bohman A. Connolly F. F. (2020). A new approach to the study of tolerance: conceptualizing and measuring acceptance, respect, and appreciation of difference. Soc. Indic. Res. 147, 897–919. doi: 10.1007/s11205-019-02176-y

[ref31] Inglehart R. Welzel C. (2005) Modernization, cultural change and democracy. New York, NY: Cambridge University Press.

[ref32] Kim S. H. Shanahan J. (2003). Stigmatizing smokers: public sentiment toward cigarette smoking and its relationship to smoking behaviors. J. Health Commun. 8, 343–367. doi: 10.1080/10810730305723 12907400

[ref33] Kleinmann A. Hall-Clifford R. (2009). Stigma: a social, cultural and moral process. J. Epidemiol. Community Health 63, 418–419. doi: 10.1136/jech.2008.084277 19439576

[ref34] Lavack A. M. (1999). De-normalization of tobacco in Canada. Soc. Mark. Q. 5, 82–85. doi: 10.1080/15245004.1999.9961068

[ref35] Link B. C. Phelan J. C. (2001). Conceptualizing stigma. Annu. Rev. Sociol. 27, 363–385. doi: 10.1146/annurev.soc.27.1.363

[ref36] Lipperman-Kreda S. Antin T. M. J. Hunt G. P. (2019). The role of multiple social identities in discrimination and perceived smoking-related stigma among sexual and gender minority current or former smokers. Drugs 26, 475–483. doi: 10.1080/09687637.2018.1490391, PMID: 34262244 PMC8276780

[ref37] Lupton D. (1995) The imperative of health: public health and the regulated body. London: Sage.

[ref38] Machado N. M. Richter K. P. Formagini T. D. B. Ronzani T. M. (2018). A qualitative review of tobacco research related to public and structural stigma. Stigma Health 3, 395–405. doi: 10.1037/sah0000110

[ref39] Major B. O’Brian L. T. (2005). The social psychology of stigma. Annu. Rev. Psychol. 56, 393–421. doi: 10.1146/annurev.psych.56.091103.070137 15709941

[ref40] Malone R. E. Grundy Q. Bero L. A. (2012). Tobacco industry denormalisation as a tobacco control intervention: a review. Tob. Control. 21, 162–170. doi: 10.1136/tobaccocontrol-2011-050200, PMID: 22345240 PMC3362192

[ref41] Marron D. O. (2017). Smoke gets in your eyes: what is sociological about cigarettes? Sociol. Rev. 65, 882–897. doi: 10.1111/1467-954X.12404

[ref42] McCool J. Hoek J. Edwards R. Thomson G. Gifford H. (2013). Crossing the smoking divide for young adults: expressions of stigma and identity among smokers and nonsmokers. Nicotine Tob. Res. 15, 552–556. doi: 10.1093/ntr/nts136, PMID: 22949576

[ref43] Mill J. S. (1974) On liberty. Harmondsworth: Penguin Books.

[ref44] Moore R. S. (2005). The sociological impact of attitudes toward smoking: secondary effects of the demarketing of smoking. J. Soc. Psychol. 145, 703–718. doi: 10.3200/SOCP.145.6.704-718 16334515

[ref45] Morphett K. Herron L. Gartner C. (2020). Protectors or puritans? Responses to media articles about the health effects of e-cigarettes. Addict. Res. Theory 28, 95–102. doi: 10.1080/16066359.2019.1596259

[ref46] Muggli M. E. Lockhart N. J. Ebbert J. O. Jimenez-Ruiz C. A. Miranda J. A. R. Hurt R. D. (2010). Legislating tolerance: Spain's national public smoking law. Tob. Control. 19, 24–30. doi: 10.1136/tc.2009.031831, PMID: 19850551

[ref47] Parsons T. (1970) The social system. London: Routledge & Kegan Paul Ltd.

[ref48] Peretti-Watel P. Legleye S. Guignard R. Beck F. (2014). Cigarette smoking as a stigma: evidence from France. Int. J. Drug Policy 25, 282–290. doi: 10.1016/j.drugpo.2013.08.009, PMID: 24315503

[ref49] Pescosolido B. A. Martin J. (2015). The stigma complex. Annu. Rev. Sociol. 41, 87–116. doi: 10.1146/annurev-soc-071312-145702, PMID: 26855471 PMC4737963

[ref50] Rao D. Angell B. Lam C. Corrigan P. (2008). Stigma in the workplace: employer attitudes about people living with HIV/AIDS in Beijing, Hong Kong, and Chicago. Soc. Sci. Med. 67, 1541–1549. doi: 10.1016/j.socscimed.2008.07.024, PMID: 18760869 PMC2740497

[ref1002] Rawls J. (1999) A theory of justice. Revised edition. Oxford: Oxford University Press.

[ref51] Ritchie D. Amos A. Martin C. (2010). ‘But it just has that sort of feel about it, a leper’ – stigma, smoke-free legislation and public health. Nicotine Tob. Res. 12, 622–629. doi: 10.1093/ntr/ntq058, PMID: 20453042

[ref52] Rokeach M. (1973) The nature of human values. New York, NY: Free Press.

[ref53] Rozin P. Singh L. (1999). The moralization of cigarette smoking in the United States. J. Consum. Psychol. 8, 321–337. doi: 10.1207/s15327663jcp0803_07

[ref54] Sæbø G. (2017). Cigarettes, snus and status: differences in lifestyle of different tobacco user groups. Health Sociol. Rev. 26, 175–189. doi: 10.1080/14461242.2016.1197043

[ref55] Sæbø G. Lund M. (2020). Are smoking cessation behaviours among daily smokers associated with a perceived public stigma of smokers? Cross-sectional analyses of Norwegian data 2011–2013. J. Smok. Cessat. 15, 189–197. doi: 10.1017/jsc.2020.25

[ref56] Sæbø G. Scheffels J. (2017). Assessing notions of denormalization and renormalization of smoking in light of e-cigarette regulation. Int. J. Drug Policy 49, 58–64. doi: 10.1016/j.drugpo.2017.07.026 28987929

[ref57] Scheffels J. Schou K. C. (2007). To be one who continues to smoke: construction of legitimacy and meaning in young adults' accounts of smoking. Addict. Res. Theory 15, 161–176. doi: 10.1080/16066350601179464

[ref58] Schwartz S. H. (2012). An overview of the Schwartz theory of basic values. Online Read. Psychol. Cult. 2, Article 11. doi: 10.9707/2307-0919.1116

[ref59] Stewart-Knox B. J. Sittlington J. Rugkasa J. Harrisson S. Treacy M. Abaunza P. S. (2005). Smoking and peer groups: results from a longitudinal qualitative study of young people in Northern Ireland. Br. J. Soc. Psychol. 44, 397–414. doi: 10.1348/014466604X18073, PMID: 16238846

[ref60] Stuber J. Galea S. Link B. G. (2008). Smoking and the emergence of a stigmatised social status. Soc. Sci. Med. 67, 420–430. doi: 10.1016/j.socscimed.2008.03.010, PMID: 18486291 PMC4006698

[ref61] Stuber J. Galea S. Link B. G. (2009). Stigma and smoking: the consequences of our good intentions. Soc. Serv. Rev. 83, 585–609. doi: 10.1086/650349

[ref62] Thompson L. Pearce J. Barnett J. R. (2007). Moralising geographies: stigma, smoking islands and responsible subjects. Area 39, 508–517. doi: 10.1111/j.1475-4762.2007.00768.x

[ref63] Tokle R. Pedersen W. (2019). ‘Cloud chasers’ and ‘substitutes’: E-cigarettes, vaping subcultures and vaper identities. Sociol. Health Illn. 41, 917–932. doi: 10.1111/1467-9566.12854 30677161

[ref64] Verkuyten M. Kollar R. (2021). Tolerance and intolerance: cultural meanings and discursive usage. Cult. Psychol. 27, 172–186. doi: 10.1177/1354067X20984356

[ref65] Weber M. (2002) The protestant ethic and the ‘spirit’ of capitalism and other writings. Harmondsworth: Penguin Books.

[ref66] Yang L. H. Kleinman A. Link B. G. Phelan J. C. Lee S. Good B. (2007). Culture and stigma: adding moral experience to stigma theory. Soc. Sci. Med. 64, 1524–1535. doi: 10.1016/j.socscimed.2006.11.013, PMID: 17188411

